# A double feedback loop mediated by microRNA-23a/27a/24-2 regulates M1 *versus* M2 macrophage polarization and thus regulates cancer progression

**DOI:** 10.18632/oncotarget.6284

**Published:** 2015-11-02

**Authors:** Sisi Ma, Min Liu, Zhenbiao Xu, Yanshuang Li, Hui Guo, Yehua Ge, Yanxin Liu, Dexian Zheng, Juan Shi

**Affiliations:** ^1^ National Laboratory of Medical Molecular Biology, Institute of Basic Medical Sciences, Chinese Academy of Medical Sciences & Peking Union Medical College, Beijing, China

**Keywords:** tumor associated macrophage, miRNA, macrophage polarization, tumor therapy, tumor microenvironment

## Abstract

In response to microenvironmental signals, macrophages undergo different types of activation, including the “classic” pro-inflammatory phenotype (also called M1) and the “alternative” anti-inflammatory phenotype (also called M2). Macrophage polarized activation has profound effects on immune and inflammatory responses, but mechanisms underlying the various types of macrophage is still in its infancy. In this study, we reported that M1-type stimulation could down-regulate miR-23a/27a/24-2 cluster transcription through the binding of NF-κB to this cluster's promoter and that miR-23a in turn activated the NF-κB pathway by targeting A20 and thus promoted the production of pro-inflammatory cytokines. Furthermore, STAT6 occupied the miR-23a/27a/24-2 cluster promoter and activated their transcription in IL-4-stimulated macrophages. In addition, miR-23a in turn suppressed the JAK1/STAT-6 pathway and reduced the production of M2 type cytokines by targeting JAK1 and STAT-6 directly, while miR-27a showed the same phenotype by targeting IRF4 and PPAR-γ. The miR-23a/27a/24-2 cluster was shown to be significantly decreased in TAMs of breast cancer patients, and macrophages overexpressing the miR-23a/27a/24-2 cluster inhibited tumor growth *in vivo*. Taken together, these data integrated microRNA expression and function into macrophage polarization networks and identified a double feedback loop consisting of the miR-23a/27a/24-2 cluster and the key regulators of the M1 and M2 macrophage polarization pathway. Moreover, miR-23a/27a/24-2 regulates the polarization of tumor-associated macrophages and thus promotes cancer progression.

## INTRODUCTION

In terms of both phenotype and function, macrophages display remarkable heterogeneity; two well-established polarized phenotypes are often referred to as the classically activated macrophages (M1 macrophages) and the alterna­tively activated macrophages (M2 macrophages) [1]. M1 macrophages, activated by interferon (IFN)-γ or other microbial products, produce large amounts of pro-inflammatory cytokines, express high levels of major histocompatibility complex molecules and are potent killers of pathogens and tumor cells [2], while M2 macrophages, usually activated by the IL-4/IL-13 immune complex, IL-10, or TGF-β, are associated with an immunosuppressive phenotype, an enhanced release of anti-inflammatory cytokines, and the abilities to support tissue remodeling and repair and to promote tumor growth and invasion [3-5]. Polarization of macrophage function is a simplified conceptual framework that is useful for describing a continuum of functional states. However, unlike TH1 and TH2 cells, M1 and M2 macrophages are not stably differentiated subsets and can switch between forms [6] in response to stimuli, a process that involves a very complicated and sophisticated mechanism and gene expression regulation. The STAT family, the nuclear receptor PPAR-γ, the CREB-C/EBP axis, the interferon regulatory factors, and the NF-κB family all participate in the regulation of polarization, involving many signaling pathways including JAK/STAT, JNK, PI3K/AKT, and Notch, among others [7-16]. Although these data provide proof of principle that different phenotypes of macrophages play key roles in the microenvironment, the regulatory mechanisms controlling the expression of genes related to the response of macrophages to activating conditions are not fully defined.

The hallmarks of cancer, which have given us a more comprehensive overview of the disease than 10 years earlier, emphasize the importance of the tumor microenvironment (TME) during every stage of tumorigenesis and invasion [17]. Of all the types of cells in the TME, macrophages seem particularly unusual. Originally, the commonly held view was that tumor associated macrophages (TAMs) should have an obvious antitumor effect, either by direct killing of tumor cells or by presenting tumor-related antigens to the body's immune system to induce tumor removal, although emerging studies have described their function in other contexts. TAMs coexist in tumors and function as an accomplice in the promotion of tumor progression, especially after being programmed and polarized into a proangiogenic/immune-suppressive (M2-like) phenotype by the TME. In this case, TAMs represent an ideal therapeutic target for blocking tumor progression after being re-programmed and re-polarized to express a pro-immune (M1-like) phenotype [1, 18].

MicroRNAs (miRNAs) are small, non-coding RNAs that influence diverse biological functions through the repression of target genes at the post-transcriptional level [19]. Growing evidence has shown that miRNAs provide functions that are essential for normal development and cellular homeostasis and, accordingly, dysfunction of these molecules has been linked to many human diseases [20]. Like transcriptional repressors, miRNAs are likely embedded in a large number of gene regulatory networks in which certain miRNA-containing circuits may be recurrent. MiRNAs also play a central role in balancing the immune response initiated upon tissue damage or pathogen recognition, and the failure miRNA-induced regulation is related to the development of inflammatory diseases [21, 22]. A number of studies implicated different miRNAs in the responses of human monocytes/macrophages in to inflammatory stimuli [23-31]. MiRNA-27a was reported to be a responsive regulator of the M1 and M2b (a name that reflects a role in antigen presentation inducing Th2 differentiation) phenotype miRNA [32]. Additionally, miR-27a regulates the inflammatory response of macrophages by targeting IL-10 [33], indicating that miR-27a, a member of the miR-23a/27a/24-2 cluster, may play an important role in macrophage polarization. However, limited in-depth data are available on the functional role of this cluster in macrophage polarization.

The miR-23a∼27a∼24-2 cluster is found to have altered expression in many diseased states. This cluster has been shown to be functional in contrasting phenotypes in different cell types [34, 35]. Several studies have also linked the expression of this cluster to cell cycle, proliferation, differentiation [36-38], thereby indicating that miR-23a∼27a∼24-2 cluster controls several processes during health and diseases. It is well documented that all three miRNAs of this cluster are derived from a single primary transcript but depending on different biological conditions their expression pattern varies [39].

In this study, we reported that miR-23a/27a/24-2, a critical miRNA cluster, could be regulated by both M1 and M2 cytokines and in turn could simultaneously regulate M1 and M2 polarization through a negative feedback loop. Furthermore, expression of the miR-23a/27a/24-2 cluster was shown to be significantly decreased in TAMs of breast cancer patients and macrophages overexpressing the miR-23a/27a/24-2 cluster showed an anti-tumor function *in vivo*. These data integrated miRNA expression and function into macrophage polarization networks and proposed a double feedback loop consisting of the miR-23a/27a/24-2 cluster and the key regulators of the M1 and M2 macrophage polarization pathway. Together, these findings aid in the exploration of potential of therapies targeting miRNAs capable of regulating TAMs phenotype switching, resulting in remodeling of the TME.

## RESULTS

### The miR-23a/27a/24-2 cluster is down-regulated by M1-type stimulation and up-regulated by M2-type stimulation

**Figure 1:**
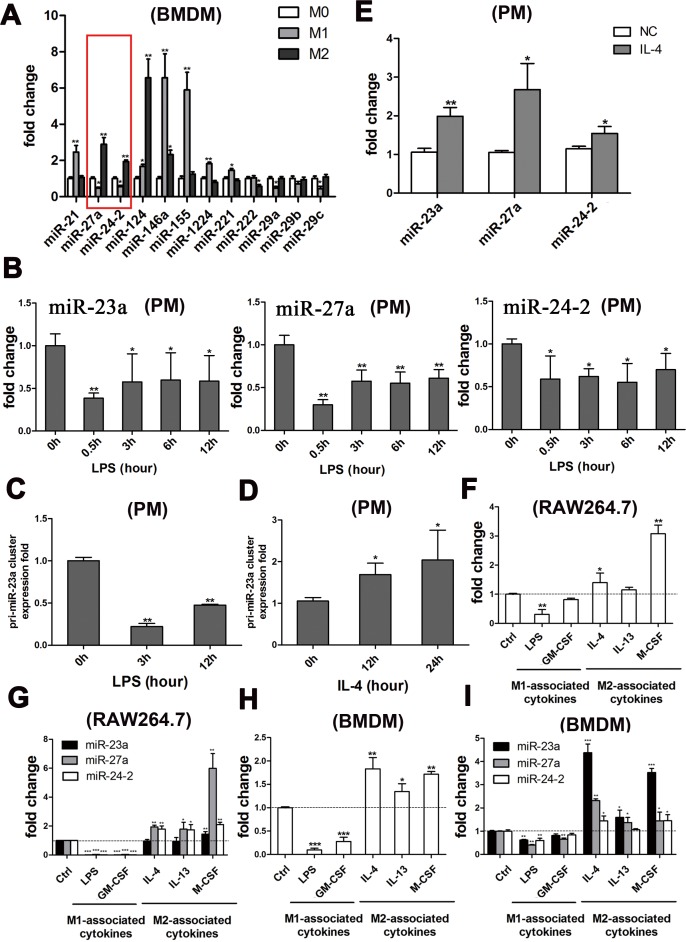
The miR-23a/27a/24-2 cluster was simultaneously down-regulated by M1-type stimuli and up-regulated by M2-type stimuli **A.** qRT-PCR analysis of the relative expression of the miRNAs in BMDMs treated with PBS (M0), 1 μg/ml LPS plus 20 ng/ml IFN-γ (M1), or 100 ng/ml IL-4 (M2). **B.** qPCR analysis of the relative expression of the mature miRNAs after the PM cells were stimulated with 1 μg/ml LPS for 0.5 h, 3 h, 6 h and 12 h. **C.** qPCR analysis of the relative expression of the cluster precursor after the PM cells were stimulated with 1 μg/ml LPS for 3 h and 12 h. **D.** qPCR analysis of the relative expression of the cluster precursor after the PM cells were stimulated with100 ng/ml IL-4 for 12 h and 24 h. **E.** qPCR analysis of the relative expression of the mature miRNAs after the PM cells were stimulated with 100 ng/ml IL-4 for 24 h. **F.** qPCR analysis of the relative expression of the cluster precursor after the RAW264.7 cells were stimulated with 1 μg/ml LPS, 20 ng/ml GM-CSF, 100 ng/ml IL-4, 60 ng/ml IL-13, or 20 ng/ml M-CSF. **G.** qPCR analysis of the relative expression of the mature miRNAs after the RAW264.7 cells were stimulated with 1 μg/ml LPS, 20 ng/ml GM-CSF, 100 ng/ml IL-4, 60 ng/ml IL-13, or 20 ng/ml M-CSF. **H.** qPCR analysis of the relative expression of the cluster precursor after the BMDMs were stimulated with 1 μg/ml LPS, 20 ng/ml GM-CSF, 100 ng/ml IL-4, 60 ng/ml IL-13, or 20 ng/ml M-CSF. **I.** qPCR analysis of the relative expression of the mature miRNAs after the BMDMs were stimulated with 1 μg/ml LPS, 20 ng/ml GM-CSF, 100 ng/ml IL-4, 60 ng/ml IL-13, or 20 ng/ml M-CSF. Mean±SD were obtained from three independent experiments. *, *p* < 0.05; **, *p* < 0.01.

We previously identified systemic variations in the expression levels of miRNAs between peritoneal macrophages and TAMs from mouse xenograft tumors (the microarray data have been deposited in NCBI Gene Expression Omnibus and are accessible through the GEO Series accession number GSE67408). The threshold value used to screen differentially expressed miRNAs was a fold change of ≥2.0, a P-value of less than 0.01, and a normalized signal value, indicating the relative abundance to the transcript, of ≥8.0. MiR-1224 was up-regulated in TAMs, while miR-146a, miR-221, miR-222, miR-29a/b/c, miR-24, and miR-21 were down-regulated in TAMs. We speculated that these miRNAs might play roles in M1 or M2 polarization thus impacting TAM differentiation. Among them, miR-146a and miR-21 were reported to be regulators of M1 or M2 macrophage polarization [40, 41], similar to miR-155, miR-124, and miR-27a [42, 43, 44]. Therefore, we detected by quantitative reverse transcriptase PCR (qRT-PCR) the expression levels of miR-1224, miR-146a, miR-221, miR-222, miR-24-2, miR-29a/b/c, miR-21, miR-155, miR-124, and miR-27a in Bone Marrow Derived Macrophages (BMDMs) that were stimulated with 100 ng/ml LPS and 20 ng/ml IFN-γ (for M1 polarization), or 100 ng/mL IL-4 (for M2 polarization) (Figure 1A). Consistent with the previous reports, expression of miR-146a, miR-21, and miR-155, important regulators of the innate immune response, was increased after LPS and IFN-γ challenge. In addition, miR-124, which was reported to be differentially expressed during macrophage polarization, was increased in IL-4-treated BMDMs. However, among the miRNAs detected, only miR-27a and miR-24-2 showed completely different variation trends with M1 *versus* M2 polarization stimuli, indicating that they may participate in both M1 and M2 macrophage polarization, thus indicating this cluster might be functionally important for the regulation of macrophage polarization and balancing the M1/M2 ratio.

To further validate the expression of the miR-23a/27a/24-2 cluster during macrophage polarization, the levels of miRNAs in peritoneal macrophages (PMs) challenged with specific stimuli were measured with qRT-PCR. As expected, when PMs were stimulated with LPS, expression levels of all three mature miRNAs were decreased (Figure 1B), similar to the precursor (Figure 1C). Moreover, the levels of the precursor of this cluster (Figure 1D) and the mature miRNAs (Figure 1E) were all markedly increased after stimulation with IL-4.

As previously reported, macrophages are polarized to the M1 phenotype by exposure to Th1 cytokines such as IFN-γ and GM-CSF, or by the presence of bacterial products such as LPS. M2 macrophages are polarized by stimulation with Th2 cytokines such as IL-4 and IL-13, as well as M-CSF [1]. To further investigate whether the miR-23a/27a/24-2 cluster could be regulated in all M1 and M2 models rather than only LPS-induced or IL-4-induced, we tested their expression levels in the RAW264.7 cell line (Figure 1F and 1G) and BMDMs (Figure 1H and 1I), treated with either M1-type stimuli, LPS, GM-CSF, or M2-type stimuli, IL-4, IL-13, M-CSF. As shown in Figure 1, expression of the precursors (Figure 1F and 1H) and the three mature miRNAs (Figure 1G and 1I) were all decreased by M1-associated cytokine stimulation and increased by M2-associated cytokine stimulation, which provided further evidence of their participation in both M1 and M2 polarization.

### NF-κB binds the promoter of the miR-23a/27a/24-2 cluster thus repressing its expression and STAT6 binds to the cluster promoter thus promoting its expression

**Figure 2:**
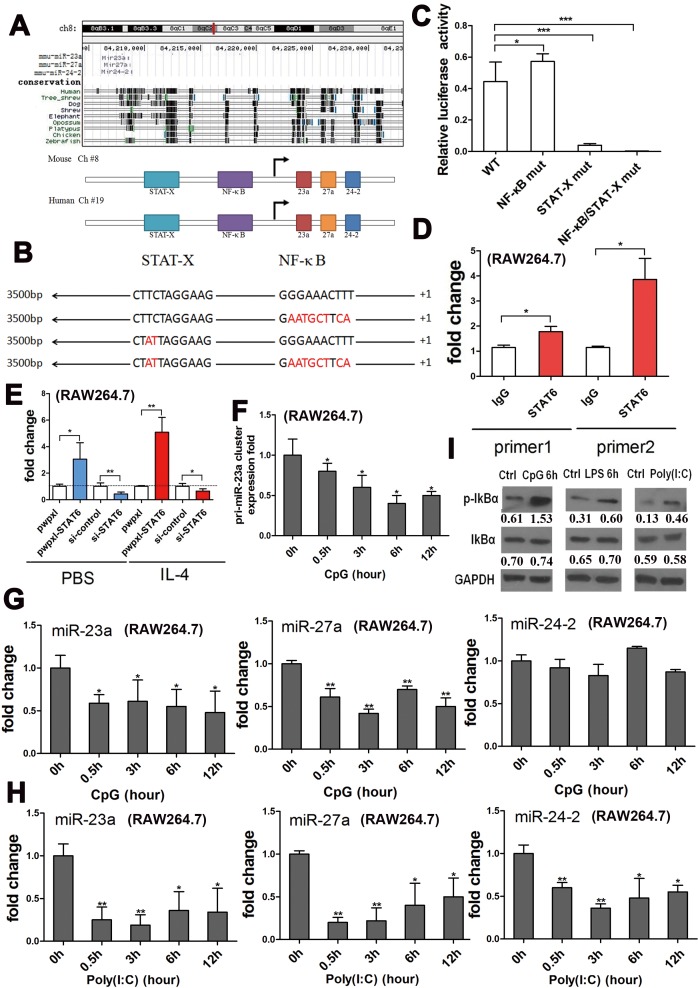
IL-4 promoted transcription of the cluster through STAT6, and LPS negatively regulated transcription of the cluster through NF-κB **A.** The miR-23a/27a/24-2 cluster was located on chromosome 8 (84208518∼ 84208921) in the genome of mus musculus (Upper panel). NF-κB and STAT-X binding sites were predicted to be located within the promoter of the cluster using bioinformatics. **B.** Wild type and three mutant promoters of the cluster were cloned into the pGL3-basic vector; the binding sites were inactivated by point mutations shown in red. **C.** Relative firefly luciferase activity derived from WT, NF-κB mut, STAT-X mut, and NF-κB/STAT-X mut constructs following transfection into 293-T cells. All values were normalized to renilla luciferase activity produced from a co-transfected control plasmid. Error bars represent standard deviations from 6 independent transfections. **D.** ChIP-qPCR assay with two different primers documenting that the TF that bound to the cluster promoter is STAT6. **E.** qPCR analysis of the cluster precursor expression in RAW264.7 cells transfected with the pwpxl-STAT6 construct or STAT6 siRNA, following stimulation with 50 ng/ml IL-4 or PBS. **F.** qPCR analysis of the relative expression of the cluster precursor after the RAW264.7 cells were stimulated with another NF-κB pathway activator, CpG, for 0 h, 0.5 h, 3 h, 6 h and 12 h. **G.** qPCR analysis of the relative expression of the three mature miRNAs after the RAW264.7 cells were stimulated with another NF-κB pathway activator, CpG OND, for 0 h, 0.5 h, 3 h, 6 h and 12 h. **H.** qPCR analysis of the relative expression of the three mature miRNAs after the RAW264.7 cells were stimulated with another NF-κB pathway activator, Poly(I:C), for 0 h, 0.5 h, 3 h, 6 h and 12 h. **I.** Western blots documenting the total IκBα and p-IκBα levels in RAW264.7 cells after stimulation with the NF-κB pathway activators, CpG OND, LPS, and Poly (I: C), normalized to GAPDH levels. Mean±SD were obtained from three independent experiments. *, *p* < 0.05; **, *p* < 0.01.

To investigate the regulation of miR-23a/27a/24-2 expression during macrophage polarization, we analyzed the promoter sequences of the cluster using a transcription element search system (http://www.cbil.upenn.edu/cgi-bin/tess). The system predicted that the binding sites for NF-κB and STAT-X, critical transcription factors in macrophage polarization, were scattered throughout the promoter region of the mouse miR-23a cluster located on Chr 8. However, in humans, the cluster is located on Chr19, and there was also a STAT-X binding site within the promoter region, while the NF-κB binding site had been previously reported [45] (Figure 2A).

To confirm the activity of the binding sites, a series of luciferase reporters containing wild type, NF-κB binding site mutated (NF-κB-M), STAT-X binding site mutated (STAT-X-M), and NF-κB/STAT-X binding sites mutated (NF-κB-M/STAT-X-M) promoters were constructed to develop a dual-luciferase assay (Figure 2B). The reporter activity was enhanced by the NF-κB binding site single mutation, reduced by the STAT-X binding site single mutation, and almost abolished by the NF-κB/STAT-X double mutation (Figure 2C), which suggested that NF-κB might suppress transcription of the cluster while STAT-X might promote its transcription. The binding site of the STAT family was highly conserved [46]; it was difficult to accurately recognize which family member it was. IL-4 could activate STAT6 during the M2 polarization pathway [1], so we employed ChIP-qPCR to confirm that STAT6 bound to the predicted site in the promoter of the miR-23a/27a/24-2 cluster (Figure 2D). Moreover, overexpression of STAT6 in the RAW264.7 cell line induced an up-regulation of the cluster precursor, and knock down of STAT6 led to a down-regulation of the precursor, regardless of the status of IL-4 stimulation. Furthermore, IL-4 stimulation partially rescued the decreased expression of the cluster precursor mediated by STAT6 knockdown (Figure 2E). Because the binding site of the STAT family was highly conserved, ChIP-qPCR with STAT1 and STAT3 antibodies was performed. The results revealed that STAT3 could also bind to the promoter while STAT1 could not (Figure S1A and S1B).

Moreover, NF-κB pathway activation induced by other TLR ligands besides LPS, such as CpG or poly (I: C), could also lead to down-regulation of the cluster precursor and of all three mature miRNAs (Figure 2F-2H). Meanwhile, the p-IκBα level was significantly enhanced by the TLR ligands, indicating an activation of the NF-κB pathway, although the total IκBα level was not increased (Figure 2I). To determine whether the miR-23a/27a/24-2 cluster expression is influenced by NF-κB activity, RAW264.7 cells were treated with LPS, CpG in the presence of the NF-κB inhibitor, BAY11-7082. As shown in Figure S1C and S1D, inhibition of NF-κB activity blocked the down-regulation of the expression of all three mature miRNAs. Thus, we concluded that IL-4 promotes the cluster transcription through STAT6-mediated positive regulation and LPS inhibits the cluster transcription through the negative regulation of NF-κB.

### Overexpression of miR-23a/27a/24-2 promotes the expression of pro-inflammatory cytokines

It was rare that both M1 and M2-associated transcription factors could simultaneously regulate the expression of the same gene. Therefore, we deduced that miR-23a/27a/24-2 must play an important role in regulating macrophage polarization or modulating M1/M2 balance.

**Figure 3:**
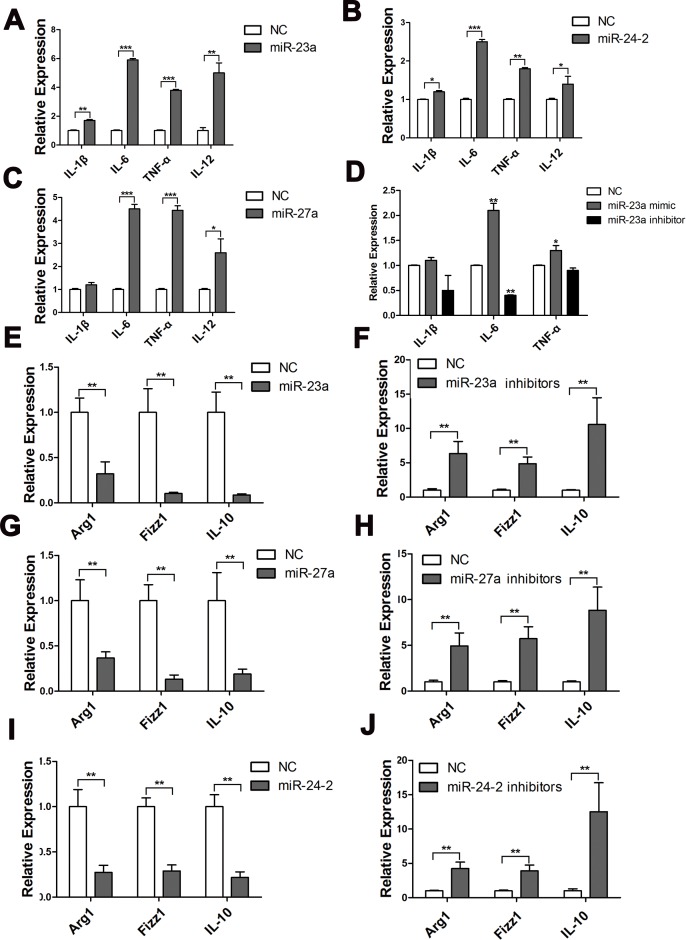
MiR-23a/27a/24-2 promoted the expression of pro-inflammatory cytokines Q-PCR analysis of the relative expression of IL-1β, IL-6, and TNF-α mRNA after RAW264.7 cells were transfected with miR-23a mimics **A.**, miR-27a mimics **B.**, or miR-24-2 mimics **C.**, for 42 h and stimulated with LPS for 6 h. **D.** qPCR analysis of the relative expression or IL-1β, IL-6, and TNF-α mRNA after PMs were transfected with miR-23a mimics or inhibitors for 42 h and stimulated with LPS for 6 h. qPCR analysis of the relative expression of Arg1, Fizz1, and IL-10 mRNA after PMs were transfected with miR-23a mimics **E.**, miR-23a inhibitors **F.**, miR-27a mimics **G.**, miR-27a inhibitors **H.**, miR-24-2 mimics **I.**, or miR-24-2 inhibitors **J.** and stimulated with IL-4. Mean±SD were obtained from three independent experiments. *, *p* < 0.05; **, *p* < 0.01.

To investigate the effect of miR-23a/27a/24-2 on the pro-inflammatory response of M1 macrophages, we treated RAW264.7 cells transfected with miR-23a/27a/24-2 mimics with LPS. The qRT-PCR analysis of the expression of the pro-inflammatory cytokines, IL-1β, IL-6, TNF-α and IL-12, showed that the overexpression of these three mature miRNAs could promote the expression of these cytokines to varying degrees (Figure 3A-3C). To further confirm this result, we then performed the same experiment in PMs and obtained similar results (Figure 3D). These data imply that the miR-23a/27a/24-2 cluster is an important regulator of the response of macrophages to M1 stimulation.

We next asked whether miR-23a/27a/24-2 also participated in macrophage plasticity by influencing the transition of macrophages to the M2 phenotype. Because IL-4 is a classic Th2 cytokine that induces M2 macrophage polarization, we evaluated the effect of miR-23a/27a/24-2 on IL-4-induced M2 polarization. PMs transfected with miR-23a/27a/24-2 mimics were stimulated with IL-4. IL-4-induced expression of Arg1, FIZZ1, and IL-10 in PMs transfected with miR-23a/27a/24-2 was significantly lower than that in PMs transfected with control mimics. Conversely, an inhibitor of miR-23a/27a/24-2 promoted the expression of Arg1, FIZZ1, and IL-10 (Figure 3E-3J). These data suggest that the miR-23a/27a/24-2 cluster is a positive regulator of pro-inflammatory responses induced by M1 stimulation in macrophages, and a negative regulator of M2 polarization.

### MiR-23a promotes M1 polarization by targeting A20 through a negative feedback loop

**Figure 4:**
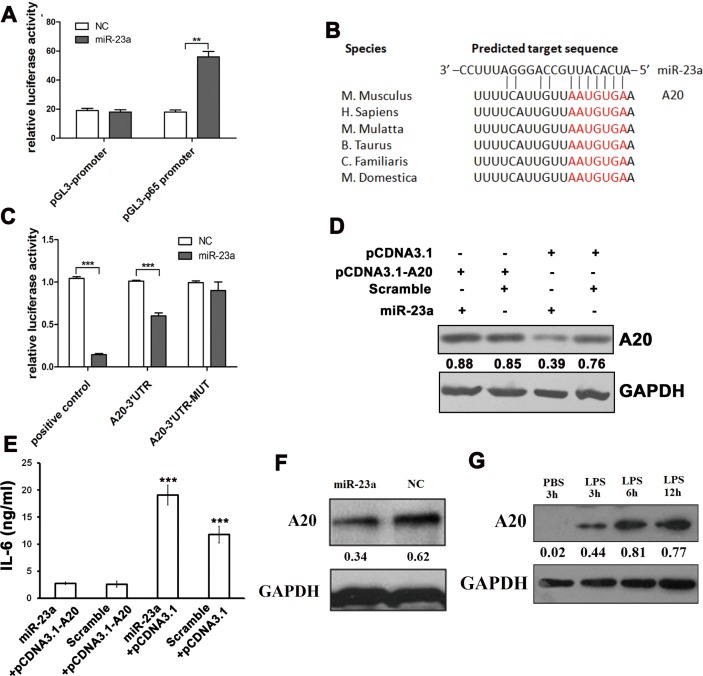
MiR-23a promoted M1 polarization by targeting A20 **A.** Relative firefly luciferase activity derived from the pGL3-p65 promoter co-transfected into 293-T cells with the TK plasmids and the miR-23a or control mimics. All values were normalized to renilla luciferase activity produced from a co-transfected control plasmid. Error bars represent standard deviations from 6 independent transfections. **B.** MiR-23a was predicted by bioinformatics to bind to the 3′UTR of A20. **C.** Relative firefly luciferase activity derived from A20-3′UTR and A20-3′UTR-MUT co-transfected into 293-T cells with the TK plasmids and the miR-23a or control mimics. All values were normalized to renilla luciferase activity produced from a co-transfected control plasmid. Error bars represent standard deviations from 3 independent transfections. The A20 protein levels **D.** and IL-6 levels in media **E.** of RAW264.7 cells transfected with pcDNA3.1-A20 for 24 hours followed by miR-23a mimics transfection. **F.** Western blots documenting the expression of A20 with PBS or 0.1 mg/ml LPS stimulation for 3 h, 6 h, 12 h in PMs, normalized to GAPDH levels. **G.** Western blots documenting the expression of A20 after transfection with miR-23a or control mimics in PMs, normalized to GAPDH levels.

The expression of pro-inflammatory cytokines was activated by NF-κB. To investigate the regulation of NF-κB by miR-23a, a pGL3-p65 promoter plasmid, containing the NF-κB promoter (NFκBαSR), was constructed to perform a dual-luciferase assay in 293T cells. The results showed that the fluorescence activity was enhanced in the miR-23a group compared with the negative control group (Figure 4A), which suggested that miR-23a activated NF-κB expression. MiRNAs always bind to the 3′UTR of their target genes to suppress target gene expression, although some can also bind to the 5′UTR or the coding region. MiR-23a simultaneously activated the NF-κB pathway and promoted the expression of M1 cytokines; therefore, the target gene of miR-23a is likely to be an upstream suppressor of the NF-κB signaling pathway. We analyzed a series of genes known to suppress the NF-κB pathway and thus identified A20, an important negative regulator of immune responses, to be a candidate target gene of miR-23a in mouse, as predicted by Targetscan algorithms [47] (Figure 4B). The wild type 3′UTR of A20 was cloned into the pMIR-Report vector. Meanwhile, a mutant form of the A20-3′UTR (A20-3′UTR-MUT), in which the miR-23a binding site was inactivated by mutating it to its complementary sequence, was constructed. Compared to the control, the luciferase activity in 293T cells co-transfected with miR-23a mimics and A20-3′UTR was significantly decreased, while mutation of the miR-23a binding site abrogated this reduction (Figure 4C). Moreover, RAW264.7 cells were transfected an A20-expressing vector lacking a 3′UTR (pcDNA3.1-A20) and then 24 hours later with transfected miR-23a mimics. The transfection of miR-23a generated a sufficient decrease in the A20 protein level. However, the miR-23a-induced decreased in A20 levels could be rescued *via* the introduction of pcDNA3.1-A20 (Figure 4D). And IL-6 level in the conditional media further demonstrated that miR-23a could not inhibit the expression of A20-expressing vector lacking a 3′UTR (Figure 4E).

Consistent with the reporter assay, the A20 protein level was decreased in the presence of miR-23a mimics (Figure 4F). As previously described, miR-23a expression is down-regulated in LPS-activated macrophages and, accordingly, the target of miR-23a is expected to be up-regulated. Indeed, as shown in Figure 4G, LPS stimulation could increase A20 expression. Taken together, these results suggest miR-23a may be among the most important molecules in regulating the inflammatory response by directly targeting A20. Furthermore, NF-κB activation represses the expression of the miR-23a/27a/24-2 cluster, forming a negative feedback loop to regulate M1 polarization.

### MiR-23a and miR-27a inhibit M2 polarization by targeting JAK1/STAT6 and IRF4/PPAR-γ, respectively, through a negative feedback loop

M1 and M2 polarizations are regulated through independent pathways; therefore, the same miRNA may target different genes in different pathways. To investigate the role of the cluster in M2 polarization, the target genes in the M2 polarization pathway were further identified. Targetscan algorithms and miRanda [48] were used to predict the target genes of miR-23a and miR-27a, and those associated with the M2 polarization pathway were selected for further analysis.

**Figure 5:**
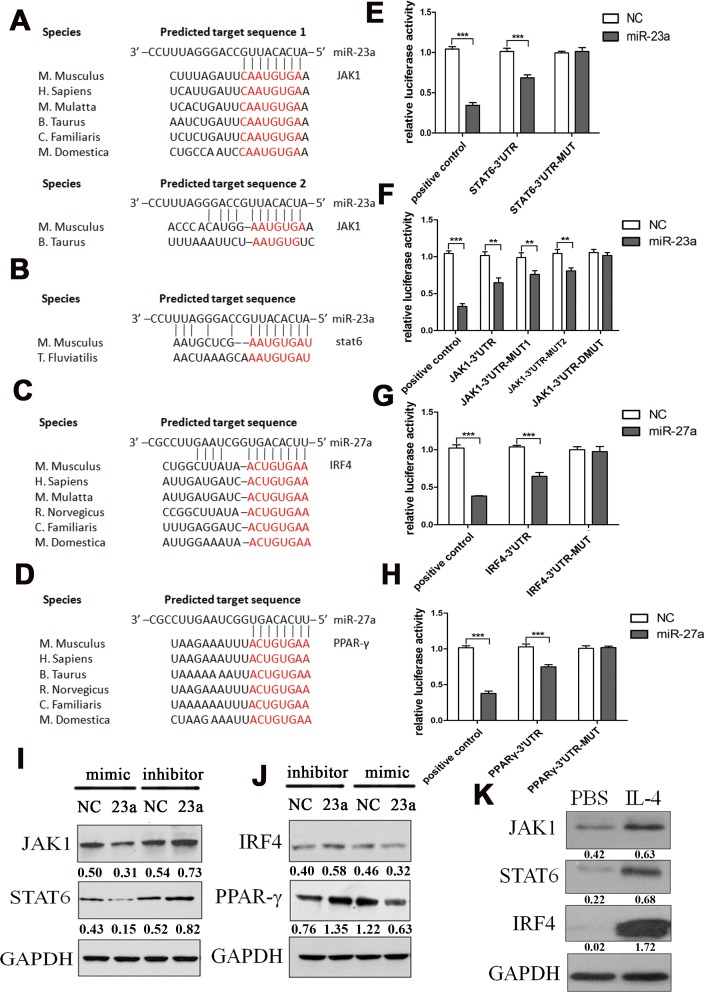
MiR-23a and miR-27a inhibited M2 polarization by targeting JAK1/STAT6 and IRF4/PPAR-γ MiR-23a was predicted by bioinformatics to bind to the 3′UTR of JAK1 at two different sites **A.** and the 3′UTR of STAT6 **B.** MiR-27a was predicted by bioinformatics to bind to the 3′UTR of IRF4 **C.** and PPAR-γ **D.** Relative firefly luciferase activity derived from STAT6-3′UTR and STAT6-3′UTR-MUT **E.**, or JAK1-3′UTR, JAK1-3′UTR-MUT1, JAK1-3′UTR-MUT2, and JAK1-3′UTR-MUT **F.** co-transfected into 293-T cells with the TK plasmids and the miR-23a or control mimics. Relative firefly luciferase activity derived from IRF4-3′UTR and IRF4-3′UTR-MUT **G.**, or PPAR-γ-3′UTR and PPAR-γ-3′UTR-MUT **H.** co-transfected into 293-T cells with the TK plasmids and the miR-27a or control mimics. Error bars represent standard deviations from 3 independent transfections, *p < 0.05, **p < 0.01, ***p < 0.001. **I.** Western blots documenting the expression of STAT6 and JAK1 after transfection with miR-23a mimics or inhibitors in PMs, normalized to GAPDH levels. **J.** Western blots documenting the expression of IRF4 and PPAR-γ after transfection with miR-27a mimics or inhibitors in PMs, normalized to GAPDH levels. **K.** Western blots documenting the expression of STAT6, JAK1, IRF4, and PPAR-γ with PBS or 0.1 mg/ml IL-4 stimulation for 12 h in PMs, normalized to GAPDH levels.

We found that JAK1 (Janus kinase 1), which is activated by ligand binding to a number of associated cytokine receptors, followed by autophosphorylation, and finally phosphorylation of their associated receptors providing multiple binding sites for signaling proteins upon cytokine receptor activation, had two potential miR-23a binding sites in its 3′UTR (Figure 5A). STAT6 (signal transducer and activator of transcription 6), which translocates to the nucleus where it regulates cytokine-induced gene expression upon activation by Janus kinases [49], was predicted to harbor a potential miR-23a binding site in its 3′UTR (Figure 5B). The IL-4 transcriptome also includes the transcription factors IRF4 (interferon regulatory factor 4), a member of the IRF family that functions with the JAK/STAT pathway [50] and harbored an miR-27a binding site in its 3′UTR (Figure 5C), as well as PPAR-γ (Figure 5D), a transcriptional activator potentially leading to an enhanced M2 phenotype in the presence of an appropriate M2 stimulus such as IL-4 [51]. To further confirm this prediction, the wild type and mutant 3′UTRs of all four genes were cloned into the pMIR-Report vector. The wild type 3′UTRs contained the binding site of miR-23a or miR-27a, while in the mutant 3′UTRs, the binding sites were inactivated by mutation to their complementary sequences. As there were two potential binding sites for miR-23a in the 3′UTR of JAK1, three mutants were constructed: binding site 1 single mutation (JAK1-3′UTR-MUT1), binding site 2 single mutation (JAK1-3′UTR-MUT2), and double mutation (JAK1-3′UTR-DMUT). Compared to the control, the luciferase activity in 293T cells co-transfected with miR-23a mimics and the STAT6-3′UTR was significantly decreased (Figure 5E). In the context of a single JAK1-3′UTR binding sites mutation, the luciferase activity was also decreased to some extent by the expression of miR-23a mimics, although less than in the wild type group, while there was no significant difference in the double mutation group (Figure 5F). The luciferase activity was also decreased with co-transfection of either miR-27a mimics/IRF4-3′UTR or miR-27a mimics/PPAR-γ-3′UTR (Figure 5G and 5H). However, respective mutations in the 3′UTRs abolished the miR-27a-induced-repression.

Then, miR-23a and miR-27a mimics and inhibitors were transfected into PMs and the expression levels of STAT6, JAK1, IRF4, and PPAR-γ were detected by western blot assay. The result showed that miR-23a mimics significantly decreased the expression of STAT6 and JAK1 and that miR-27a mimics decreased the expression of IRF4 and PPAR-γ, consistent with the miRNA inhibitor results (Figure 5I and 5J). The above results demonstrated that JAK1 and STAT6 were the target genes of miR-23a, while IRF4 and PPAR-γ were the target genes of miR-27a in M2 polarization.

To further establish the regulatory correlation between the miRNAs and their target genes, the expression levels of STAT6, JAK1, IRF4, and PPAR-γ upon IL-4 stimulation were further evaluated in PMs. As shown in Figure 5K, IL-4 increased the expression of STAT6, JAK1, IRF4, and PPAR-γ in PMs.

Taken together, these results indicate that expression of the miR-23a/27a/24-2 cluster is increased by M2-type cytokines and that miR-23a and miR-27a repress M2-associated transcription factors, forming a negative feedback loop to regulate M2 polarization.

### The miR-23a/27a/24-2 cluster is decreased in TAMs, and overexpression of the miR-23a/27a/24-2 cluster in macrophages suppresses tumor growth *in vivo*

Originally, the commonly held view was that TAMs should have an obvious antitumor effect, either by direct killing of tumor cells or by presenting tumor-related antigens to induce the body's immune response to suppress tumor growth, although emerging studies have described their function in other contexts. Different phenotypes of TAMs have different influences on tumors; for example, M1-like macrophages have an anti-tumor function while M2-like macrophages could promote tumors to some degree. The important M2-like characteristic of TAMs is their ability to influence tumor growth *via* the promotion of tumor angiogenesis, survival, and metastasis [52].

**Figure 6:**
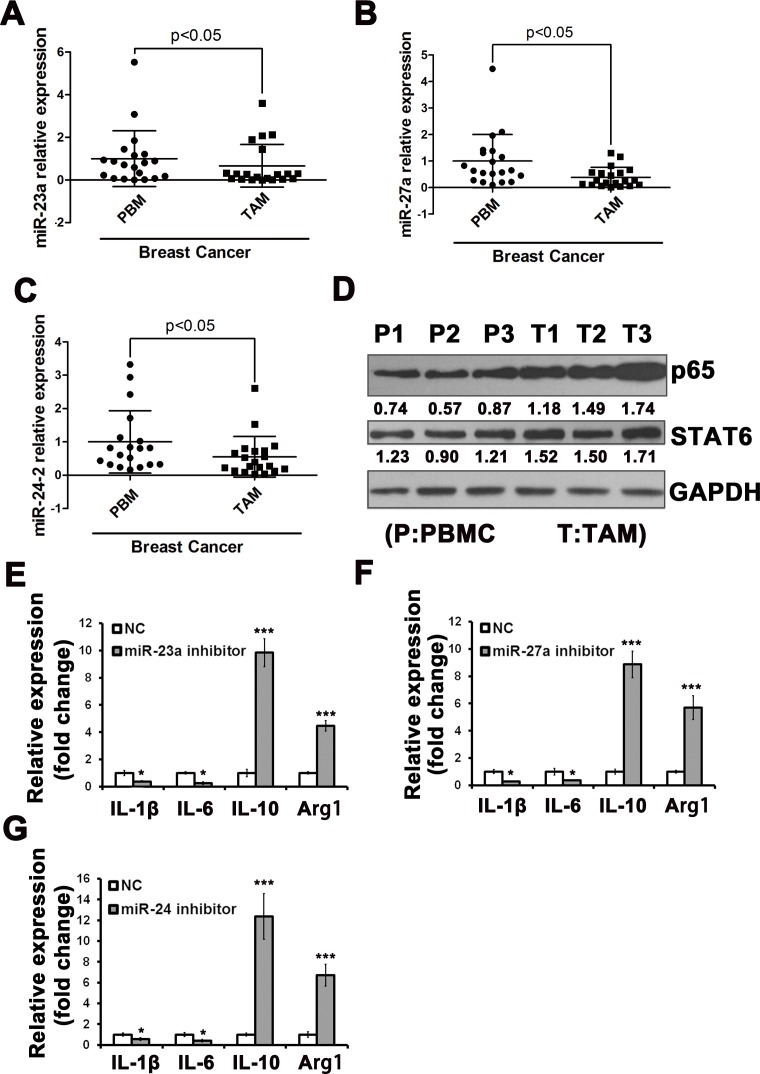
The miR-23a/27a/24-2 cluster was down-regulated in TAMs in breast cancer patients Expression level of miR-23a **A.**,miR-27a **B.**, and miR-24-2. **C.** in TAMs isolated from breast cancer tissue compared with paired PBMCs of stage II or III patients, as revealed by qRT-PCR assay (n = 20). **D.** The protein levels of NF-κB p65 subunit and STAT6 in PBMCs and TAMs. The expression levels of M1 and M2 cytokines in TAMs after transfected with miR-23a inhibitors **E.**, miR-27a inhibitors **F.** or miR-24-2 inhibitors **G.**

As a result, we focused on the expression of this cluster in TAMs from cancer patients. To investigate the expression of miR-23a/27a/24-2 in the TAMs of clinical tumor samples, we performed qRT-PCR for miR-23a/27a/24-2 in paired TAMs and peripheral blood monocyte (PBMC) samples isolated from 20 patients with breast carcinoma. We found that miR-23a/27a/24-2 expression was significantly decreased in human breast tumor TAMs compared with paired human PBMCs (Figure 6A-6C). We showed that the miR-23a/27a/24-2 cluster was down-regulated by M1-type stimulation and up-regulated by M2-type stimulation. However, it seemed paradoxical that this cluster was down-regulated in TAMs of breast cancer patients. Because the level of NF-κB p65 subunit and STAT6 is essential for their transcriptional activity, we detected the expression level of p65 and STAT6. As shown in Figure 6D, compared with PBMCs, p65 and STAT6 were both enhanced in the TAMs of clinical tumor samples.

We isolated TAMs from mouse 4T1 xenograft tumors and transfected with miRNA inhibitors and then detected the cytokines expression. The results showed that the M1 type cytokines were decreased and the M2 type cytokines were increased (Figure 6E-6G), which were consistent with the results *in vitro*.

**Figure 7:**
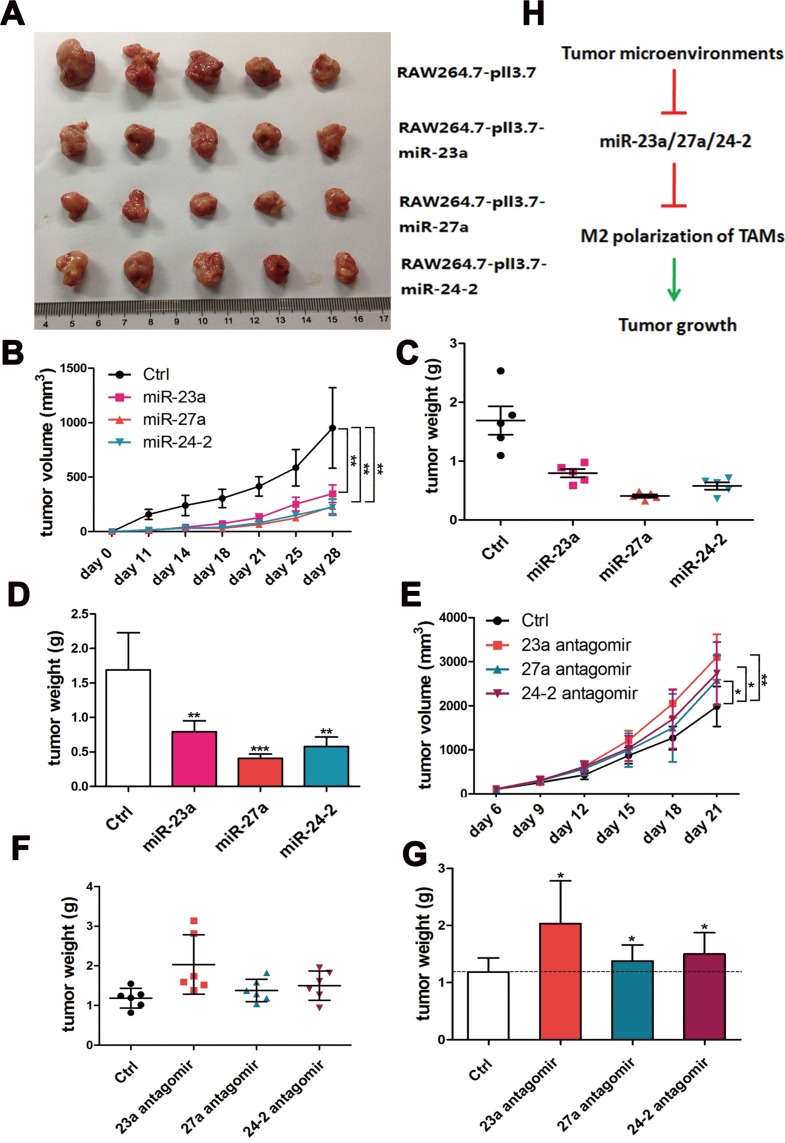
The miR-23a/27a/24-2 cluster reduced tumor growth *in vivo* Approximately 3:1 4T1 cells and RAW264.7-miR-23a/27a/24-2 cells or control cells were s.c. co-injected into 4-6 week-old BALB/c female mice. 11 days later, tumor lengths and widths were measured with a caliper every 3 or 4 days until day 28 (*n* = 5). Tumor volumes at different time points were shown in **A.** and **B.** Tumor weights and average weights at the experimental end point were shown in **C.** and **D.** Approximately 3:1 4T1 cells and BMDM-miR-23a/27a/24-2 antagomir cells or control cells were s.c. co-injected into 4-6 week-old BALB/c female mice. 6 days later, tumor lengths and widths were measured with a caliper every 3 days for 3 weeks (*n* = 5). Tumor volumes at different time points were shown in **E.** Tumor weights and average weights at the experimental end point were shown in **F.** and **G. H.** Schematic representing M2 polarization of TAMs through the regulation of the miRNA-23a cluster. ***p < 0.001; **p < 0.01; *p < 0.05.

Therefore, to investigate the function of the cluster *in vivo*, miR-23a, miR-27a, and miR-24-2 overexpression lentiviruses were packaged, and then used to infect RAW264.7 cells; a stable expression cell line was obtained using FACS. A cellular mixture of mouse breast cancer 4T1 cells and corresponding stable RAW264.7 cells at a ratio of 3:1 were s.c.-injected into BALB/c nude mice. The result showed that tumors were smaller and weighed less from the miR-23a, miR-27a, and miR-24-2 overexpression group compared to the negative control group (Figure 7A-7D). To further confirm the function of the cluster, BMDMs were transfected with miR-23a, miR-27a, and miR-24-2 antagomirs. Then, the cellular mixture of 4T1 cells and BMDMs were injected into the mice at a 3:1 ratio. The result showed that tumors were bigger and heavier from the miR-23a, miR-27a, and miR-24-2 antagomir group compared to the negative control group (Figure 7E-7G). Those results showed that the inhibition of miR-23a, miR-27a, and miR-24-2 in macrophages promotes tumor growth *in vivo*, consistent with the overexpression results.

**Figure 8:**
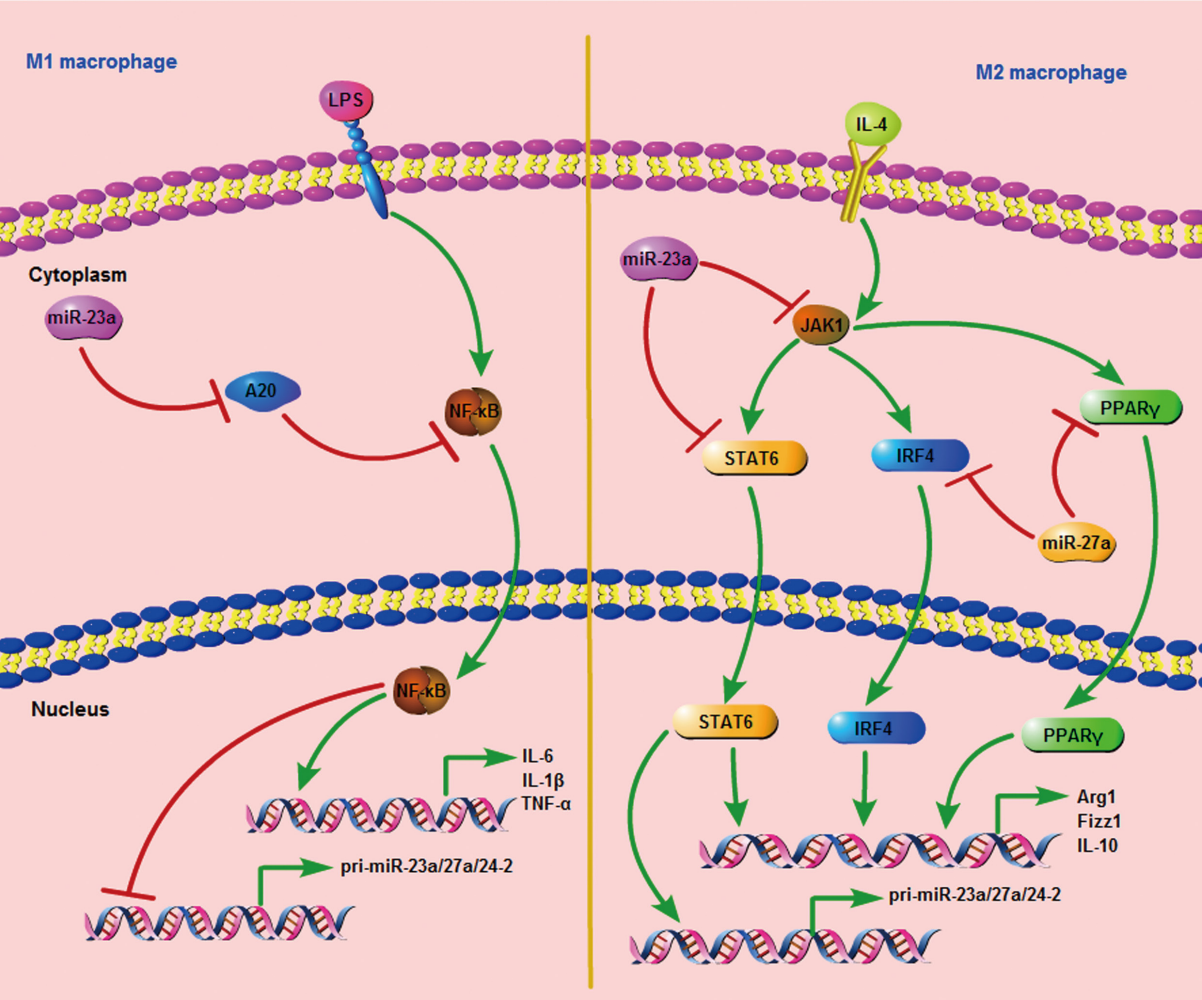
The signal pathway consists of the miR-23a cluster and important regulators during M1 and M2 polarization

Taken together, our data suggest that the miR-23a/27a/24-2 cluster is down-regulated during M1 polarization, and it promotes M1 associated cytokine production by activating the NF-κB promoter and inhibiting the NF-κB pathway inhibitor, A20, through a negative feedback loop. Additionally, STAT6 occupies the miR-23a/27a/24-2 promoter and promotes its expression during M2 polarization. Furthermore, the miR-23a/27a/24-2 cluster in turn inhibits M2 cytokine production by directly targeting JAK1 and STAT6 with miR-23a and IRF4 and PPAR-γ with miR-27a through a negative feedback loop (Figure 8).

## DISCUSSION

Overexpression of miR-23a, miR-27a, and miR-24-2 could all promote the expression of M1 cytokines and reduce the expression of M2 cytokines, suggesting that the three miRNAs play an important role in inflammation and macrophage polarization and that the miR-23a/27a/24-2 gene cluster was likely to express three types of mature miRNAs that play a greater role through a synergistic effect. Although all from the same precursor, miR-23a, miR-27a, and miR-24-2 did not always behave the same way, for example, their sensitivities to the same stimulus varied due to differential changes in their expression levels, which gave us a clue that in spite of the same transcriptional regulation, there might be post-transcriptional regulation during precursor processing. This mechanism required further investigation. Furthermore, the function of each mature miRNA also seemed to display a distinct preference. Although they collaboratively promoted the expression of M1 cytokines, overexpression of miR-23a tended to promote IL-6 production rather than IL-1β or TNF-α, while miR-27a “preferred” TNF-α. Thus, the mature miR-23a, miR-27a, and miR-24-2 transcripts might be differently regulated during precursor processing, and as a result, they seemed to play distinct roles in determining macrophage polarization.

Detailed study on the function of miR-23a during M1 polarization suggested that the target gene of miR-23a was likely to be an upstream suppressor of the NF-κB signaling pathway; therefore, we analyzed candidate genes including MyD88s, TAG, SHIP-1, SOCS3, TOLLIP, and A20, and finally confirmed that A20 (TNFAIP3) was the potential target gene of miR-23a using dual-luciferase assay and western blot. However, this was only one possible mechanism of miR-23a-mediated function, and it might also target other inflammation-related genes to regulate the NF-κB signaling pathway, and the target genes of miR-27a and miR-24-2 remain to be further investigated.

When studying the transcriptional regulation of the cluster, we predicted that NF-κB and STAT-X binding sites were scattered within its promoter region. We then confirmed the result using dual-luciferase assay by showing that when the NF-κB binding site is mutated the fluorescence is enhanced slightly, and when the STAT-X binding site was mutated the fluorescence was reduced significantly. Therefore, the fluorescence in the double mutation group should be stronger, theoretically, compared with the STAT-X single mutation group. However, intriguingly, when both of the binding sites were mutated, the fluorescence almost completely disappeared. It remains to be seen whether NF-κB and STAT6 interacted with each other in addition to independently regulating the transcription of the cluster. Ching-Hung et al. reported that the actions of NF-κB and STAT6, and their direct interaction with each other, might provide a basis for the synergistic activation of transcription by IL-4 and NF-κB activators [53]. Moreover, we also found that STAT3 could also bind to the miR-23a cluster promoter while STAT1 could not. And STAT1 is activated in response to M1 macrophage-polarizing signals whereas STAT3 are selectively activated by M2 macrophage-polarizing cytokines [54]. As a result, M1 and M2 macrophage polarization stimuli might regulate transcription of the cluster interdependently rather than independently, but this hypothesis still requires more evidence to be clarified.

We showed that the miR-23a/27a/24-2 cluster was down-regulated by M1-type stimulation and up-regulated by M2-type stimulation. However, it seemed paradoxical that this cluster was down-regulated in TAMs of breast cancer patients. Many solid tumors, including breast cancer, are composed of heterogeneous cell populations that associated in complex networks. Tumor cells also recreate complex cellular microenvironments. Nonetheless, evidence suggests that many cytokines, such as TGF- β, IL-6, IL-10, etc. exert complex effects on tumor development. Our results also suggested that NF-κB and STAT6 might interdependently regulate transcription of the cluster. Furthermore, NF-κB p65 subunit and STAT6 showed increased level in TAMs compared with PBMCs. Therefore we hypothesized that the decreased expression of the miR-23a/27a/24-2 cluster in TAMs of breast cancer patients might be related to the tumor microenvironment or tumorigenic factors including the activation balance between NF-κB and STAT6 pathway, etc. Taken together, our data demonstrate that the miR-23a/27a/24-2 cluster is a potential therapeutic target due to its ability to regulate TAM phenotype switching, resulting in the remodeling of the TME. However, the reason for its decreased expression level detected in TAMs needs to be further clarified.

In conclusion, we reported that the miR-23a/27a/24-2 cluster could be regulated by both M1 and M2 cytokines and could in turn regulate M1 and M2 polarization through a negative feedback loop. Furthermore, macrophages overexpressing the miR-23a/27a/24-2 cluster showed an anti-tumor effect *in vivo*. These data integrated miRNA expression and function into macrophage polarization networks and proposed a double feedback loop consisting of the miR-23a/27a/24-2 cluster and the key regulators of the M1 and M2 macrophage polarization pathway. Furthermore, expression of the miR-23a/27a/24-2 cluster was shown to be significantly decreased in TAMs of breast cancer patients and macrophages overexpressing the miR-23a/27a/24-2 cluster showed an anti-tumor effect *in vivo*.

## MATERIALS AND METHODS

### Cell culture, treatment and transfection

The RAW264.7 cells, mouse peritoneal macrophages (PMs), bone marrow derived macrophages (BMDMs) are cultured in RPMI 1640 supplement with 10% FBS (Hyclone), 100 U/mL penicillin and 100 g/mL streptomycin at 37°C in 5% CO_2_. The cells were stimulated with 1μg/mL LPS, 100ng/mL IL-4, 60ng/mL IL-13, 20ng/mL M-CSF/GM-CSF (Peprotech) to detect the expression of the cluster. TAMs were isolated from 47 fresh tumor samples with a Percoll Density Gradient Centrifugation kit (Pharmacia). MiR-23a/27a/24-2 mimics and inhibitors from GenePharma (Shanghai, China) were used for the overexpression and inhibition of the mature miRNAs, transfected at a final concentration of 10nM into macrophages using Lipofectamine^™^ 2000 reagent (Invitrogen) according to the manufacturer's instructions. The siRNAs of STAT6(Integrated DNA Technologies) were transfected at a final concentration of 10 nM and 1 nM using Lipofectamine^TM^ 2000 reagent for 24 and 48h. Negative controls were transfected to serve as matched controls.

### Patients and clinical samples

Breast tumor tissue samples and paired peripheral blood were collected from breast cancer 2 and 3 stage patients with tumor resection. Informed consent for the use of samples was obtained from all patients before surgery, and approval was obtained from the Chinese Academy of Medical Sciences.

### Peritoneal macrophages isolation

Female BALB/c mice (6 to 8 week) were injected intraperitoneally (i.p.) with 2 mL of sterile 3% thioglycollate (Difco, Detroit, MI, USA). Three days later peritoneal macrophages were collected by PBS intraperitoneal lavage [44]. After centrifugation the cells were resuspended in RPMI 1640 medium (Hyclone) supplemented with 10% heat-inactivated-FBS (Hyclone). Then seeded the cells in culture plates, after 2 h of incubation to allow macrophages to adhere, each well was washed three times with warm Hank's balanced salt solution medium to remove non-adherent cells.

### Bone marrow derived macrophages isolation

Bone marrow-derived macrophages grown in M-CSF (BMM) or GM-CSF (GM-BMM) were generated as described previously [55]. Briefly, bone marrow cells were isolated from the femurs of mice and cultured in RPMI 1640 (Hyclone), supplemented with 10% heat-inactivated-FBS (Hyclone) in the presence of 10ng/mL of M-CSF (Peprotech) or GM-CSF (Peprotech). At day 4, nonadherent cells were collected and cultured for a further 3 days in the presence of fresh CSF (10ng/mL). On day 7, adherent cells were collected and used in subsequent experiments [56].

### Quantitative RT-PCR assays

Total RNA was extracted from the harvested cells using Trizol reagent (Invitrogen, Carlsbad, CA, USA) according to the manufacturer's instruction. cDNA was synthesized *via* M-MLV reverse transcriptase (Promega). Oligo (dT) 15 was used as the RT primers for reverse transcription of mRNAs. MiR-23a, 27a, 24-2, U6 snRNA were reverse transcriped using specific RT primers. Quantitative RT-PCR was performed in a Applied Biosystems Step-One real-time PCR System (Applied Biosystems, Foster City, CA, USA) using the SYBR Green PCR Mix (Takara, Dalian, China) at 95°C for 10 min, followed by 40 cycles of 95°C for 15 s and 60°C for 1 min according to the manufacturer's instructions. The comparative Ct method was used to quantify the target genes relative to the endogenous control. For the mRNAs, the data were normalized with the endogenous GAPDH or beta-actin control. For the miRNAs, U6 snRNA was used as the endogenous control. All the PCR reactions were performed in triplicate. The primers were listed in Supplementary Table 1.

### Constructs and lentivirus package

The promoter of the miR-23a/27a/24-2 cluster containing NF-κB and STAT binding sites and their mutations were PCR amplified and cloned into PGL3-basic reporter vector (Promega, WI, USA) upstream of the firefly luciferase gene to generate the pGL3-23a/27a/24-2-promoter reporter. The promoter of the p65 was also PCR amplified and cloned into PGL3-basic reporter. The 3′-UTRs of mouse A20, JAK1, STAT6, IRF4 and PPAR-γ mRNAs were PCR amplified and cloned into pMIR-reporter downstream of the firefly luciferase gene to generate the corresponding reporters. All the constructed plasmids were confirmed by DNA sequencing. The lentivirus vector expressing miR-23a (pll3.7-miR-23a), miR-27a (pll3.7-miR-27a) and miR-24-2 (pll3.7-miR-24-2) were co-transfected into 293TN cell line with three helper vectors (pGag/pol, pRev and pVSV-G) for packaging. The viruses were concentrated by ultrafiltration, and then infected the RAW264.7 cell line to obtain stable expression cell line.

### Dual-luciferase assay

For the functional analysis of the miR-23a promoter, 293T cells were co-transfected with 100ng pGL3-23a promoter-WT or pGL3-23a promoter-M constructs and 20ng pRL-TK vectors per well of 96-well plate. For the functional analysis of the p65 promoter, 100 ng pGL3-p65 promoter was co-transfected with 5 pmol miRNA mimic or miR-23a and 20ng pRL-TK vectors per well of 96-well plate. For the miRNA target analysis, 293T cells were co-transfected with 100 ng reporter construct, 20 ng pRL-TK vector, and 5 pmol miRNA mimic or miR-23a per well of 96-well plate. Cells were harvested 48 h post-transfection and assayed with a Dual Luciferase Assay (Promega, WI, USA) according to the manufacturer's instructions. All transfection assays were performed in triplicate.

### Western blotting

Western blot assay was performed as described previously [49]. Protein extracts were resolved through 12% SDS-PAGE, transferred to PVDF membranes, and probed with antibodies against mouse STAT6 (CST), IRF4 (CST), NF-κB (CST), JAK (CST), A20 (CST), p65 (CST), PPAR-γ (CST) and normalized with the endogenous GAPDH control. Peroxidase-conjugated anti-mouse or rabbit IgG (CST) was used as secondary antibody and the antigen-antibody reaction was visualized by enhanced chemiluminescence assay (ECL, Thermo).

### ChIP assay

Chromatin immunoprecipitation (ChIP) assay was performed as described previously [57]. Briefly, 1×10^7^ RAW264.7 cells were cultured in 10-cm culture dishes and treated with 100 ng IL-4 for 24 h. Cells were cross-linked with 1% formaldehyde (Sigma-Aldrich) for 10 min at 37°C. The cells were then washed in cold PBS buffer, resuspended in lysis buffer (0.1% SDS, 0.5%Triton X-100, 20 mM Tris-HCl [pH 8.1], 150 mM NaCl, protease inhibitor cocktail), collected by scraping and then sonicated to yield chromatin DNA fragments ranging in size from 200 to 1000 bp, followed by centrifugation for 10 min. Ten microliters of the cleared chromatin was reserved as input DNA sample. Immunoprecipitation analysis was carried out at 4°C overnight using anti-STAT6 antibodies (CST), and anti-mouse IgG was used as a control for nonspecific binding. Immunoprecipitated DNA was analyzed by q-PCR using two pairs of promoter-specific primers of miR-23a/27a/24-2 cluster promoter both containing the STAT6 binding sites. Expression of a target DNA sequence was normalized to the input DNA and represented as fold enrichment compared with the non IL-4-treated control (set as one fold).

### Animal experiment

Female 6- to 8-week-old BALB/c mice were kept under specific pathogen-free conditions. Animal experiments proceeded in accordance with the guidelines of the National Institutes of Health. 4T1 cells and RAW264.7 cells were injected into the mice *via* the 3:1 ratio. The tumor size was monitored every 2 or 3 days for 25 days. Tumor volume (V) was calculated using the formula V = (*ab*^2^)/2, in which *a* is the longest and *b* the shortest diameter of the tumor.

### Statistical analysis

Results of quantitative data in this study are expressed as the mean ±SD. Statistical differences between groups were compared using two-tailed ANOVA *via t* test. A P value of less than .05 was considered significant (* *P*-values < 0.05, ** *P*-values < 0.01, *** *P*-values < 0.001).

## SUPPLEMENTARY MATERIAL FIGURE AND TABLE


