# Supplementary material for: A double feedback loop mediated by microRNA-23a/27a/24-2 regulates M1 versus M2 macrophage polarization and thus regulates cancer progression
Source: Oncotarget. 2015 Nov 2;7(12):13502–19. doi: 10.18632/oncotarget.6284 (PMC4924657; doi:10.18632/oncotarget.6284)
Supplement: Supplementary file 1 [file oncotarget-07-13502-s001.pdf]

A double feedback loop mediated by microRNA-23a/27a/24-2 regulates M1 *versus* M2 macrophage polarization and thus regulates cancer progression

Supplemental Material

Supplemental Table 1. Primer sequence used for qRT-PCR and constructs.

Gene	Primer Sequence
beta-actin	5'-3'CATGTACGTTGCTATCCAGGC<br>5'-3'CTCCTTAATGTCACGCACGAT
miR-23a/27a/24-2 cluster precursor	5'-3'TCTGCCGTGAGCAAGACCTGGAA<br>5'-3'GTGGACACCCAGATGCAGGCT
miR-24-2	RT5'-3'<br>GTCGTATCCAGTGCGTGTCGTGGAGTCGGCAATTG<br>CACTGGATACGACCTGTTCCT<br>5'-3'GGGGGGGTGGCTCAGTTCA<br>5'-3'CAGTGCAGGGTCCGAGGT
miR-27a	RT5'-3'<br>GTCGTATCCAGTGCGTGTCGTGGAGTCGGCAATTG<br>CACTGGATACGACAGCGGAAC<br>5'-3'GGGGGGGTTCACAGTGGCT<br>5'-3'CAGTGCAGGGTCCGAGGT
miR-23a	RT5'-3'<br>GTCGTATCCAGTGCGTGTCGTGGAGTCGGCAATTG<br>CACTGGATACGACGGAAATCC<br>5'-3'GGGGGGGATCACATTGCCA<br>5'-3'CAGTGCAGGGTCCGAGGT
miR-146a	RT5'-3'<br>GTCGTATCCAGTGCGTGTCGTGGAGTCGGCAATTG<br>CACTGGATACGACAACCCATG<br>5'-3'GGGGGGTGAGAACTGAAT

	5’-3’CAGTGCAGGGTCCGAGGT
miR-155	RT5’-3’<br>GTCGTATCCAGTGCGTGTCGTGGAGTCGGCAATTG<br>CACTGGATACGACACCCCTAT<br>5’-3’GGGGGGGTTAATGCTAATT<br>5’-3’CAGTGCAGGGTCCGAGGT
miR-124	RT5’-3’<br>GTCGTATCCAGTGCGTGTCGTGGAGTCGGCAATTG<br>CACTGGATACGACGGCATTCA<br>5’-3’GGGGGGGTAAGGCACGCGG<br>5’-3’CAGTGCAGGGTCCGAGGT
miR-21	RT5’-3’<br>GTCGTATCCAGTGCGTGTCGTGGAGTCGGCAATTG<br>CACTGGATACGACTCAACATC<br>5’-3’GGGGGGGTAGCTTATCAGA<br>5’-3’CAGTGCAGGGTCCGAGGT
U6	RT 5’-3’CGCTTCACGAATTTGCGTGTCAT<br>5’-3’GCTTCGGCAGCACATATACTAAAAT<br>5’-3’CGCTTCACGAATTTGCGTGTCAT
IL-1 β	5’-3’ ATCTCGCAGCAGCACATC<br>5’-3’CAGCAGGTTATCATCATCATCC
IL-6	5’-3’ CAGAAGGAGTGGCTAAGGACCA<br>5’-3’ ACGCACTAGGTTTGCCGAGTAG
TNF- α	5’-3’ TGACAAGCCTGTAGCCCACG<br>5’-3’ GACTCCAAAGTAGACCTGCCCG
IL-12	5’-3’ATCTACCGAAGTCCAATGCAA<br>5’-3’ATTTCAACAGCATAAGGCCAA
Arg-1	5’-3’CTTGGCTTGCTTCGGAACTC<br>5’-3’GGAGAAGGCGTTTGCTTAGTTC

Fizz1	5’-3’ACTCGTTGACTGGACCACTG<br>5’-3’AAGAAGCAGGGTAAATGGGCA
IL-10	5’-3’CATACTGCTAACCGACTCCT<br>5’-3’CTCCACTGCCTTGCTCTTA
Primer1(ChIP)	5’-3’GGAGTTTCCTCCTGCCCTTC<br>5’-3’TCTGCTTCTATCCCCACCCA
Primer2(ChIP)	5’-3’ATGGGGAAACTGAGGCTTGG<br>5’-3’GCCATGGAGAGGAAGCTGAG
pll3.7-miR-23a	5’-3’ AAAAGTTAACAGGCAGGCAAGCAAGAATGC<br>5’-3’ CCGCTCGAG GGCTTCTCTGTTACCCAGTACCC
pll3.7-miR-27a	5’-3’ AAAAGTTAACGTGCTCTGCCTTGGGGGCTC<br>5’-3’ CCGCTCGAGAGCCCTGCCAGCCAGGAGG
pll3.7-miR-24-2	5’-3’ AAAAGTTAACCATCTCCTCAGGCCGCTGCT<br>5’-3’ CCGCTCGAGGATGCAGGCTTAGAGGCTGAGC
cluster promoter wild type	5’-3’ CGACGCGTGCCACCAACTGCAACTGTTGTAGT<br>5’-3’ CCGCTCGAGCCATGGAGAGGAAGCTGAGGG
NF-κB mut	5’-3’ CGACGCGTCCTAGGAATGCTTCAACTGAGGTG<br>5’-3’ CCGCTCGAGCCATGGAGAGGAAGCTGAGGG
stat6-mut	5’-3’ CGACGCGTGCCACCAACTGCAACTGTTGTAGT<br>5’-3’ CCGCTCGAGTGGCGCCACTTCCTAATAGC
NF-κB/stat6 mut	5’-3’ CGACGCGTCCTAGGAATGCTTCAACTGAGGTG<br>5’-3’ CCGCTCGAGTGGCGCCACTTCCTAATAGC
P65 promoter	5’-3’ GGGGCTAGCCTGGTCTACATATTG<br>5’-3’ ATTATCCCGTTCCCCGCCCC
A20 3’UTR	5’-3’ GGACTAGTGAGGAAGTTTCTGCCACTGC<br>5’-3’CGACGCGTTTGTTCTTAAATAATTTATTTTTTAAT<br>A
A20 3’UTR-MUT	5’-3’ TCATTGTTGGCGCGGAACGGTTATTTATA-CTTA<br>5’-3’TAACCGTTCCGCGCCAACAATGAAAATCAATAG

Stat6 3’UTR	5’-3’ GCCTTCCGTCTCAACTGTTCC<br>5’-3’ TCTGGGTAGAACTCACACCAGTGA
Stat6<br>3’UTR-MUT	5’-3’GGGTTAATGCTCGTTACACTT<br>5’-3’ TCTGGGTAGAACTCACACCAGTGA
JAK1 3’UTR	5’-3’ GTTGACTTGGAGGTAGCTGGGT<br>5’-3’ AGGAAGCAGTGGAGGGAACTT
JAK1<br>3’UTR- MUT1	5’-3’TAGATTGTTACACTACCGCAGA<br>5’-3’AGGAAGCAGTGGAGGGAACTT
JAK1<br>3’UTR- MUT2	5’-3’GTTGACTTGGAGGTAGCTGGGT<br>5’-3’GACACTAGTGTAAGGTACTGGGTCC
JAK1<br>3’UTR-DMUT	5’-3’TAGATTGTTACACTACCGCAGA<br>5’-3’GACACTAGTGTAAGGTACTGGGTCC
IRF4 3’UTR	5’-3’TTGAAGACAACAACAGGGCTTTG<br>5’-3’TTTACTTCCCTGAGAAATGGACCA
IRF4<br>3’UTR-MUT	5’-3’CCCTGGCTTATATGACACTAAC<br>5’-3’TTTACTTCCCTGAGAAATGGACCA
PPAR-γ 3’UTR	5’-3’CAGGAAAGTCCCACCCGCTG<br>5’-3’GGGGGGGGAATTTTATAATGTGG
PPAR-γ<br>3’UTR-MUT	5’-3’TAAGAAATTTTGACACTAAAAG<br>5’-3’GGGGGGGGAATTTTATAATGTGG

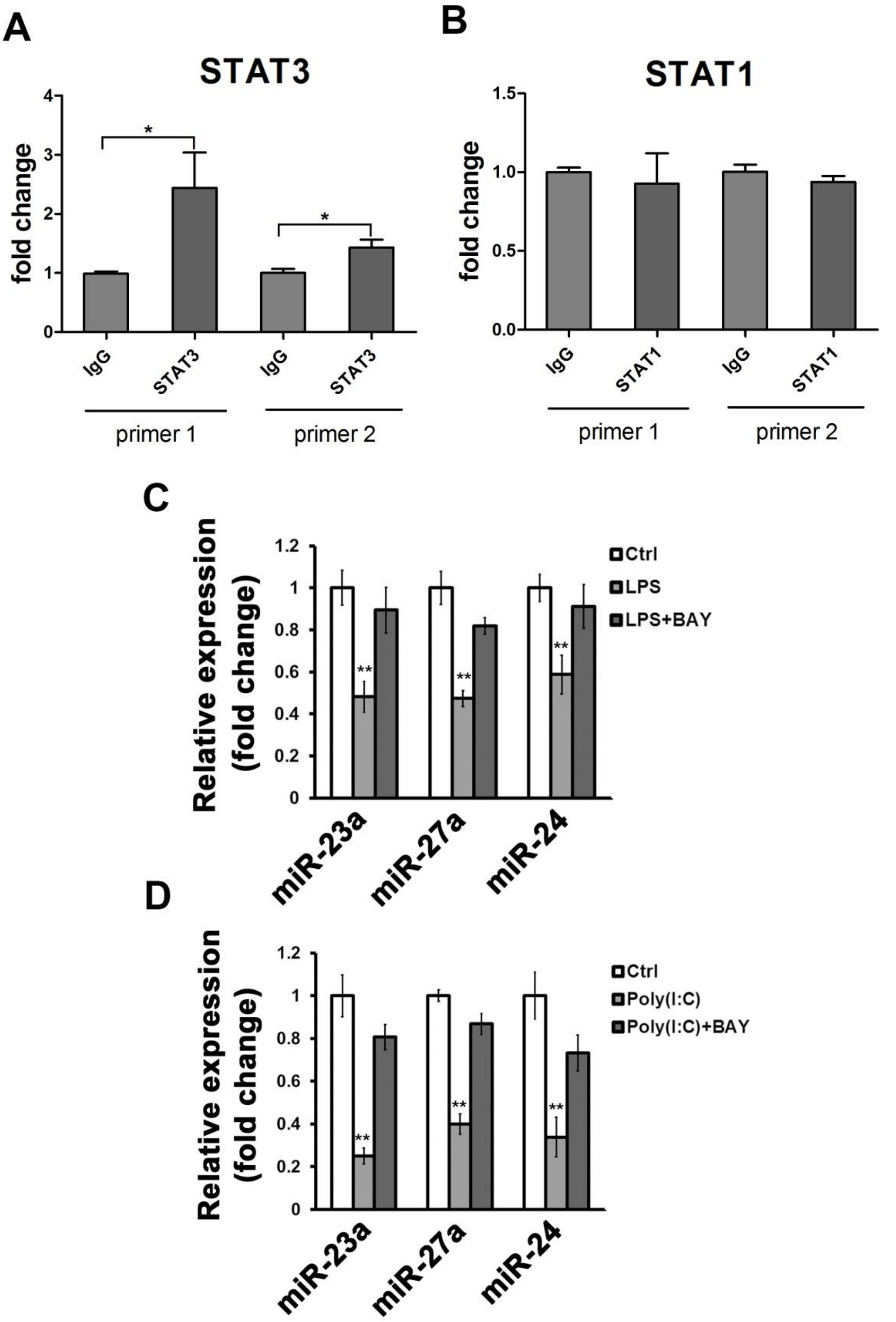

Figure S1. NF-κB and STAT family could regulate the cluster transcript. ChIP-qPCR assay using two different primers documenting that the transcription factor STAT3 could bind to the cluster's promoter in IL-4-stimulaiton **(A)** wihle STAT1 could not **(B)**. The NF-κB inhibitor, BAY11-7082, could block the down-regulation of the expression of all three mature miRNAs induced by LPS **(C)** and poly (I: C) **(D)**.
